# Enzymatically Hydrolyzed Amaranth as a Base for Enteral Nutrition: Nutritional Characterization, Phenolic Bioaccessibility, and Physicochemical Properties

**DOI:** 10.3390/foods15173028

**Published:** 2026-08-27

**Authors:** Dina Khamitova, Saule Saduakhasova, Dana Toimbayeva, Zhanna Kamirova, Neslihan Taş, Vural Gökmen, Svetlana Kamanova, Kadyrzhan Makangali, Nurlan Dautkanov, Gulnazym Ospankulova

**Affiliations:** 1 Department of Food Technology and Processing Products, Technical Faculty, Saken Seifullin Kazakh Agrotechnical University, Zhenis Avenue, 62, Astana 010000, Kazakhstan; dina.khamitova@nu.edu.kz (D.K.); saulesaduahasova5@gmail.com (S.S.); bio.dana@mail.ru (D.T.); kamizhanna@mail.ru (Z.K.); kamanovasveta@mail.ru (S.K.); k.makangali@kazatu.edu.kz (K.M.); 2 Department of Biology, School of Sciences and Humanities, Nazarbayev University, Kabanbay Batyr 53, Astana Z05H0P9, Kazakhstan; 3 Regional Technological Park QazAgroOrganic LLP, Zhenis 62b, Astana Z11F9K5, Kazakhstan; 4 Food Quality and Safety (FoQuS) Research Group, Department of Food Engineering, Hacettepe University, Ankara 06800, Turkey; neslihangoncuoglu@hacettepe.edu.tr (N.T.); vgokmen@hacettepe.edu.tr (V.G.); 5 Aron R&D Co. LLP, Akmeshit Street, 5, Astana 010000, Kazakhstan; 6 Department of Crop Production Processing, Kazakh Research Institute of Processing and Food Industry, Almaty 050060, Kazakhstan

**Keywords:** amaranth, enteral nutrition, enzymatic hydrolysis, plant-based, INFOGEST

## Abstract

Plant-based enteral formulas represent a hypoallergenic alternative to dairy-derived products, but incorporation of underutilized pseudocereals into enteral formulas remains insufficiently characterized. This study developed a laboratory-scale prototype of a fortified beverage for enteral administration (FBEA) from the soluble fraction of *Amaranthus hypochondriacus*, with added pea protein isolate, safflower oil, and carbohydrate components. Sequential enzymatic treatment with α-amylase and Alcalase hydrolyzed amaranth grain flour into soluble fraction, increasing free amino acid content relative to the unhydrolyzed flour, including arginine from 0.52 to 241.39 mg kg^−1^, lysine from 3.91 to 129.10 mg kg^−1^, and glutamic acid from 103.62 to 368.54 mg kg^−1^. Fortification yielded a protein content of 25.46% DW, a lipid content of 24.95% DW, and an energy density of 1014 kcal L^−1^, consistent with isocaloric formulas, with seven essential amino acids meeting FAO/WHO/UNU reference adequacy values. In vitro digestion showed that the soluble fraction and FBEA exceeded amaranth flour in bioaccessibility of antioxidant activity and phenolic content, with FBEA achieving the highest intestinal phenolic release. The formula met ESPEN physicochemical recommendations, with osmolality at 292 mOsm kg^−1^ and viscosity at 51 mPa·s, and simulated tube flowability comparable to two commercial formulas. These findings indicate that enzymatically hydrolyzed amaranth shows potential as a plant-based enteral formulation prototype, though clinical suitability requires further safety, shelf-life, and tolerance data.

## 1. Introduction

Global demand for functional plant-based foods and beverages has grown substantially over the past decade, driven by the rising prevalence of chronic diseases, consumer interest in healthier options, and sustainability concerns [1]. Moreover, fortified functional food products are becoming increasingly recognized as a relevant component in therapeutic nutrition for vulnerable populations, including elderly patients and individuals with gastrointestinal disorders [2].

Enteral nutrition (EN) is a key method of nutritional support for patients with a functional gastrointestinal tract and insufficient oral intake [3]. Besides its macronutrient composition, clinical applicability of EN formulas depends on physicochemical criteria including energy density, osmolality, viscosity, and stability during administration [4]. Another significant factor is digestibility and nutrient bioavailability, since impaired absorption increases complication risks, including diarrhea and bloating [5]. Therefore, the development of an EN formula requires consideration of both nutritional quality and physicochemical functionality.

Despite these requirements, most commercial EN products rely on dairy-derived proteins such as casein and whey or their hydrolysates [6]. Lactose intolerance affects an estimated 65% of the global adult population [7], and IgE-mediated cow’s milk protein allergy represents a notable contraindication for both pediatric and tube-fed patients [8]. In addition, the livestock-derived ingredient supply chain generally carries a higher environmental burden relative to plant-based alternatives [9]. These limitations have stimulated interest in plant-derived proteins as alternative ingredients for specialized nutrition products. However, replacing animal-derived proteins with plant protein isolates does not necessarily address all technological challenges associated with the formulation of nutritionally complete EN products, particularly those related to viscosity, osmolality, suspension stability, digestibility, and tube administration.

Plant protein sources represent a promising basis for specialized nutrition products. Among them, amaranth (*Amaranthus* spp.) stands out as a gluten-free pseudocereal with high nutritional and functional value. It is characterized by a well-balanced amino acid profile with high lysine content and contains significant levels of dietary fiber, as well as bioactive compounds such as squalene and tocopherols, which exhibit strong antioxidant properties [10]. However, the intact grain contains a complex matrix of proteins, starch, lipids, fiber, and phenolic compounds that may contribute to its nutritional and functional value while simultaneously limiting water solubility, suspension stability, and rheological behavior suitable for beverage-type formulations [10,11]. To overcome these limitations, biotechnological processing methods such as enzymatic hydrolysis can be applied. Enzymatic degradation of starch can reduce viscosity through the formation of lower-molecular-weight carbohydrates, while proteolysis can improve protein solubility through the generation of peptides and free amino acids. Hydrolytic processing may also facilitate the release of matrix-associated phenolic compounds and thereby modify the antioxidant properties of the resulting fraction [12]. However, whether these modifications can simultaneously provide the nutritional, physicochemical, and technological characteristics required for an enteral nutrition formulation remains insufficiently characterized.

Previous studies on amaranth have focused mainly on bakery products, fermentation processes, protein isolates, and bioactive peptide hydrolysates with antioxidant and antihypertensive activities [13,14]. In contrast, existing plant-based enteral nutrition formulas are largely based on isolated proteins, particularly soy or pea protein, and are primarily positioned as alternatives to dairy-dominant protein blends [15].

To the best of our knowledge, no previous study has evaluated the soluble fraction of an enzymatically hydrolyzed whole-grain amaranth flour as the primary functional base of a multicomponent enteral nutrition formulation, particularly with respect to its combined nutritional composition, physicochemical properties, tube administration performance, and in vitro gastrointestinal digestibility. Unlike formulations based on isolated plant proteins, a soluble whole-grain hydrolysate retains hydrolyzed proteins alongside soluble carbohydrates and water-soluble bioactive compounds, while enzymatic processing is intended to improve protein solubility and reduce the viscosity associated with intact starch. The potential advantage of this approach therefore lies not simply in replacing animal-derived proteins with plant-based ingredients, but in integrating the nutritional and bioactive components of the whole grain with a processing strategy intended to improve technological functionality.

Because the suitability of an enteral formulation cannot be determined from nutritional composition alone, an integrated analytical strategy was adopted, combining nutritional, physicochemical, technological, and in vitro digestion perspectives within a single experimental framework. We hypothesized that sequential enzymatic hydrolysis of whole-grain amaranth flour with α-amylase and protease, followed by fortification with plant-based ingredients, would improve the technological functionality of the resulting formulation—specifically, by reducing starch-related viscosity and increasing protein solubility.

The aim of this study was to develop and experimentally characterize a plant-based enteral nutrition formula based on the soluble fraction of an enzymatic hydrolysate of whole-grain amaranth flour (*Amaranthus hypochondriacus*). To achieve this aim, the following objectives were addressed: (i) to characterize the nutritional composition and free amino acid profile of the developed formulation using proximate analysis, in comparison with unhydrolyzed amaranth flour; (ii) to determine its viscosity, osmolality, and pH stability and assess these parameters against relevant reference ranges for enteral nutrition formulations; (iii) to evaluate tube administration performance and compare the physicochemical properties of the developed formulation with commercial enteral nutrition products; and (iv) to assess antioxidant activity and total phenolic content before and after standardized in vitro gastrointestinal digestion using the INFOGEST 2.0 static digestion protocol.

## 2. Materials and Methods

### 2.1. Description of Materials and Chemicals

Amaranth (*Amaranthus hypochondriacus*) was cultivated and obtained from farmers in the Petropavlovsk region of Kazakhstan. For enzymatic hydrolysis, commercial preparations of α-amylase BAN 480 L and Alcalase^®^ 2.4 L FG (Novozymes, Beijing, China) were used. According to the manufacturer’s technical specifications, the activity of α-amylase BAN 480 L was 480 KNU-B/g at a preparation density of 1.25 g/mL, while the activity of Alcalase^®^ 2.4 L FG was 2.4 AU-A/g at a density of 1.17 g/mL. Mineral and vitamin premixes were obtained from Del’Ar (Krasnogorsk, Russia). Pea protein was acquired from 100ING (Petersburg, Russia). Lecithin was procured from Lasenor (Olesa de Montserrat, Spain) and safflower oil was obtained from Kaz-Ir Agro (Birlik, Zhambyl region, Kazakhstan). Maltodextrin was acquired from NewBio LLC (Volgograd, Russia). The stabilizer mix was purchased from Cis-market (Almaty, Kazakhstan). The mineral premix, vitamin premix, pea protein isolate, and stabilizer mix used in this formulation were food-grade ingredients intended for dietary supplement applications. Detailed composition, as provided by the manufacturers, is presented in Table 1, and food safety certificates were available from the manufacturers upon request.

Potassium chloride, potassium dihydrogen phosphate, sodium bicarbonate, sodium chloride, sodium hydroxide, magnesium chloride hexahydrate were purchased from Merck (Darmstadt, Germany); ammonium carbonate, hydrochloric acid, and calcium chloride dihydrate used in the standardized INFOGEST protocol were obtained from Sigma-Aldrich (St. Louis, MI, USA). The digestive enzymes applied in the analytical experiment included salivary α-amylase (cat.no. 1031), α-amylase from porcine pancreas (cat.no. A3176), pepsin from porcine gastric mucosa (cat.no. P7012), bovine bile (cat.no. B3883), lipase from porcine pancreas (cat.no. L0382), trypsin (cat.no. T4799) and colipase (cat.no. C3028) from porcine pancreas, and α-chymotrypsin from bovine pancreas (cat.no. C4129), all bought from Sigma-Aldrich (St. Louis, MI, USA). Chemical reagents for analytical experiments were also obtained from Sigma-Aldrich (St. Louis, MI, USA).

Commercial enteral products “Nutrien Standard” (isocaloric, 4.0 g protein per 100 mL, Infraprim LLC, Moscow, Russia) and “Nutrison” (isocaloric, 5.5 g protein per 100 mL, Nutricia, The Netherlands) were purchased from a local pharmacy in Astana, Kazakhstan, for comparison of nutritional composition and evaluation of tube performance.

### 2.2. Preparation of Amaranth Flour

The grain was stored under dry conditions at 22.5 ± 0.5 °C until processing. Prior to the experiments, the amaranth grains were washed and dried in a drying oven at 45 °C for 12–16 h until a moisture content of 4.5 ± 0.5% was reached, as measured using a Radwag MA 50/1 moisture analyzer (Radom, Poland). The dried grain was milled into flour using a laboratory mill (LZM, Moscow, Russia). During milling, the temperature was controlled at 40 °C to prevent denaturation of proteins and preserve bioactive compounds. The resulting amaranth grain flour (AGF) was sieved through a 1 mm mesh.

### 2.3. Enzymatic Hydrolysis and Plant-Based Fortification

Distilled water with amaranth flour at a ratio of 1:5 was transferred to a preheated food mixer Thermomix TM7 (Vorwerk, Wuppertal, Germany) and mixed at 1700× *g* until formation of a homogeneous mixture. In the first stage, α-amylase BAN 480 L was added at a dosage corresponding to 0.60 KNU-B/g of amaranth flour and incubated at 65 °C for 1 h. In the second stage, Alcalase^®^ 2.4 L FG was added at a dosage of 0.0281 AU-A/g of amaranth flour, and incubation was continued at 55 °C for 1 h. The pH of the suspension was monitored without active adjustment throughout the two-stage enzymatic hydrolysis. The initial flour–water suspension had a pH of 6.8 ± 0.03, which decreased to 6.7 ± 0.05 following α-amylase treatment and further to 6.3 ± 0.02 following Alcalase treatment. The effect of controlled pH adjustment on hydrolysis efficiency was not evaluated in this study and is identified as a direction for future optimization. At the end of hydrolysis, enzymes were thermally inactivated by heating the mixture to 90 °C for 10 min.

The resulting hydrolysate was transferred into sterile containers and rapidly cooled in an ice bath. The samples were centrifuged using a CM-7S Plus centrifuge (ELMI, Riga, Latvia) at 1 100× *g* for 15 min, followed by supernatant collection and storage at 4 °C until further analysis. Hydrolysis efficiency was assessed by the soluble solids yield of the hydrolysate, calculated as the ratio of freeze-dried supernatant dry mass to the initial dry mass of amaranth flour, which was 80.0%.

The additive levels shown in Table 1 were selected based on the nutritional targets of the formulation, the contribution of the hydrolyzed amaranth suspension (AHS), and the need to obtain a physically stable product with the desired energy density and osmolality. The formulation was designed to provide approximately 1000 kcal/L, with target levels of 50 g/L protein, 50 g/L fat, and 90 g/L carbohydrates, in accordance with the relevant nutritional recommendations for enteral nutrition [21,22,23]. Pea protein isolate was added as the protein-density contributor, complementing the amaranth-derived protein fraction. Safflower oil and lecithin were combined to establish the lipid phase as a stable oil-in-water emulsion at the target fat content. Maltodextrin was included as a low-osmolality carbohydrate source, allowing the target energy density to be reached without disproportionately raising osmolality. The stabilizer mix was added to maintain emulsion and suspension stability across the formulation steps. Mineral and vitamin premix levels follow the manufacturer’s recommended dosage for an isocaloric beverage matrix and were not independently re-optimized for this formulation.

Supernatant from amaranth hydrolysate (AHS) was transferred into the food mixer preheated to 45 °C. After that, mineral and vitamin premix were added to supernatant and mixed at 300× *g* for 4 min. Then, pea protein isolate was introduced into the reactor and homogenized at 2000× *g* for an additional 4 min.

In parallel, two separate solutions were prepared using magnetic stirrers MS-01 (ELMI, Riga, Latvia). Maltodextrin was dissolved in distilled water at a ratio of 1:1 until complete dissolution. Separately, the oil phase was prepared by mixing safflower oil and lecithin in the ratio specified in Table 1 until a homogeneous emulsion with a stable foam layer was obtained. Both solutions were added to the food reactor together with stabilizer mix and homogenized further at 6150× *g* for 10 min to ensure complete homogenization.

The control sample (CSA) was prepared according to Table 1, without amaranth hydrolysate, using distilled water preheated to 45 °C in place of AHS, followed by the identical fortification stages, mixing speeds, and durations described above.

Where required, samples were lyophilized prior to analysis using a HyperCool 3110 freeze-drying system (Hanil, Gimpo, Republic of Korea) at −50 °C and 0.05 mbar until constant weight was achieved. Lyophilized samples were subsequently ground to a fine homogeneous powder and stored at −20 °C in sealed containers until further analysis.

### 2.4. Nutritional Content Analysis

Protein content was determined using the Kjeldahl method [24]. Samples were digested to convert organic nitrogen into ammonium ions using a Kjeldahl digestion block (Gerhardt, Königswinter, Germany), followed by distillation (Gerhardt, Königswinter, Germany) and titration using a digital titrator (VWR, Radnor, PA, USA) to determine total nitrogen, calculated by factor 6.25.

Total fat content was determined using the Soxhlet extraction method [25] using an automated Soxhlet system (Gerhardt, Königswinter, Germany) with hexane as the solvent.

Ash content was determined using a modified AOAC 942.05 method [26]. Prior to combustion, samples were charred and then incinerated in a muffle furnace (SNOL, Narkunai, Lithuania) at 650 °C for 1 h until complete combustion of organic matter.

Total carbohydrate content was determined using the anthrone–sulfuric acid method [27]. Briefly, 1 mL of diluted sample was mixed with 4 mL of freshly prepared anthrone reagent in an ice bath. The mixture was then heated in a boiling water bath at 100 °C for 10 min and subsequently cooled rapidly to room temperature. Absorbance of mixture was measured spectrophotometrically at 620 nm on UV-1900i Plus spectrophotometer (Shimadzu, Kyoto, Japan). A standard calibration curve was constructed using glucose solutions in the concentration range of 0–100 μg/mL (R^2^ = 0.999) to determine equivalent content in sample mixtures.

Total dietary fiber content was determined using the enzymatic–gravimetric method [28]. Samples diluted in phosphate buffer (pH 6.0) were sequentially treated with α-amylase, protease, and amyloglucosidase to remove digestible components. The remaining residue was precipitated with ethanol and acetone, filtered, dried, and weighed for gravimetric determination of dietary fiber content, subtracting the mass of ash and protein determined according to [26] and [25], respectively.

As protein, fat, and total dietary fiber were determined according to established AOAC methods [25,26,29], the precision and accuracy of these methods follow their published AOAC validation parameters. All nutritional composition analyses were performed using independently prepared samples from three separate experimental preparations (*n* = 3 independent replicates). Each analytical measurement was performed in triplicate as technical measurements, and the mean of the technical measurements was used as one observation for statistical analysis.

*Energy value* was calculated using Atwater factors [29], which assign energy values based on macronutrient composition:

(1) $$Energy\;value,\;kcal\;=\;\left(A\;*\;4\right)+(B\;*\;9)+\;\left(C\;*\;4\right)$$

where A—total protein quantity in grams; B—total fat content in grams; C—total carbohydrates content in grams.

### 2.5. Analysis of Amino Acid Profiles

Determination of total and free amino acids was performed according to the method of Göncüoğlu Taş and Gökmen [30] and Yilmaz et al. [31], respectively, with minor modifications. For complete amino acid analysis, a weighed sample was placed in glass tubes, 5 mL of 8 N HCl was added, the headspace was flushed with nitrogen, and the tubes were hermetically sealed. Hydrolysis was carried out at 110 °C for 23 h. After hydrolysis, the samples were filtered, and 100 μL of the filtrate was collected, evaporated under a stream of nitrogen, and reconstituted in 1 mL of water, followed by adjustment to 80% acetonitrile.

For determination of free amino acids, 1 g of dry sample underwent three-stage extraction with water (10, 5, and 5 mL). Samples were vortexed, centrifuged at 4650× *g* for 3 min, and the supernatants were collected. The extracts were then combined with 800 µL of ACN, and the resulting supernatant was collected. Prior to analysis, samples were filtered through a 0.45 μm nylon filter. Finally, 450 µL of filtered sample was mixed with 500 µL of acetonitrile–water mixture (80:20 *v*/*v*) and 50 µL of internal standard in autosampler vials.

Amino acid determination was performed using UPLC-MS/MS (Waters, Milford, MA, USA). Separation was carried out on a Merck ZIC-HILIC column (150 × 4.6 mm, 3.5 μm) using a gradient of water (0.1% formic acid, phase A) and acetonitrile (0.1% formic acid, phase B) at a flow rate of 1 mL/min and a column temperature of 30 °C. The elution gradient was as follows: 80% B (0–3 min), linear decrease to 40% B (3–5 min), hold at 40% B (5–8 min), return to 80% B (8–9 min), and re-equilibration to 15 min. Electrospray source settings were as follows: cone voltage, 15 V; capillary voltage, 2.5 kV; source temperature, 150 °C; desolvation temperature, 600 °C; desolvation gas (nitrogen) flow, 1000 L/h; cone gas flow, 50 L/h. Detection was performed in positive ionization mode using multiple reaction monitoring (MRM) transitions. Calibration was carried out using standard amino acid solutions in the range of 0.1–2.0 mg/L. LOD and LOQ values for amino acids ranged from 0.5–5 and 1.7–16.7 μg/kg, respectively. Amino acid extraction and UPLC-MS/MS analysis were performed for each of the three independently prepared samples (*n* = 3), with each extract injected once.

### 2.6. In Vitro Digestibility of Samples

In vitro simulated gastrointestinal digestion of the AGF, AHS, CSA and FBEA was performed according to the harmonized INFOGEST 2.0 static protocol described by Brodkorb et al. [32] without matrix specific modification. Prior to each digestion experiment, the activities of all digestive enzymes were determined experimentally for each new batch according to the standardized assays provided in the protocol. Simulated salivary fluid (SSF, pH 7.0), simulated gastric fluid (SGF, pH 3.0), and simulated intestinal fluid (SIF, pH 7.0) were prepared at 1.25× electrolyte stock concentrations according to Table 3 of Brodkorb et al. [32] and pre-warmed to 37 °C before use.

The oral phase was applied only to AGF, which required reconstitution to a swallowable bolus consistency prior to simulated oral digestion, per INFOGEST 2.0 recommendations for high-solid foods. The oral phase was omitted for AHS, CSA and FBEA, since FBEA is intended for administration via feeding tube, bypassing oral processing in its real route of use, and AHS and CSA were digested under matched conditions to preserve comparability across the AGF-AHS-CSA-FBEA series.

The samples were subjected to sequential gastric and intestinal digestion phases, with pH adjustments performed as required at each stage. Digestion was carried out at 37 °C using an orbital shaker–incubator ES-20/60 (BioSan, Riga, Latvia). Samples were collected after 1 h and 2 h of both the gastric and intestinal digestion phases.

Enzyme activities were terminated at each sampling time point by heat inactivation at 90 °C for 5 min. Samples were then centrifuged at 5000× *g* for 10 min at 4 °C, and the supernatants were collected and frozen immediately at −20 °C. For each digestion phase, enzyme-blank controls were run in parallel under identical conditions, and their readings were subtracted from the corresponding sample values to eliminate background contributions from enzyme autolysis products, bile salts, and reagents in the TPC and radical-scavenging activity assays. Three independent digestion experiments were performed on separately prepared samples (*n* = 3 independent replicates), each using its own digestion vessel. For each independent batch, three analytical measurements were performed on the resulting digesta; these technical replicates were averaged prior to statistical analysis.

### 2.7. Determination of Bioaccessibility After In Vitro Digestion

#### 2.7.1. Total Phenolic Content

Total phenolic content (TPC) was determined using the Folin–Ciocalteu method according to Singleton et al. [33]. Samples were diluted with methanol (1:4, *v*/*v*). An aliquot of 1 mL of each diluted sample was mixed with 1 mL of Folin–Ciocalteu reagent and incubated at room temperature for 5 min. Subsequently, 1 mL of 7.5% (*w*/*v*) sodium carbonate solution was added. Reaction mixtures were incubated at room temperature for 1 h, and absorbance was measured at 765 nm on the spectrophotometer described in Section 2.4. Total phenolic content was quantified using a gallic acid calibration curve (y = 0.0019x + 0.1274, R^2^ = 0.999) and expressed as mg gallic acid equivalents (GAE) per liter.

#### 2.7.2. DPPH and ABTS Radical Scavenging Activity

DPPH and ABTS radical scavenging activity was evaluated according to Cuvas-Limon et al. [34] with slight modifications. A 0.4 mM DPPH solution was prepared in methanol. Fourfold diluted supernatant from the samples (0.1 mL) was mixed with 3.9 mL of DPPH solution and incubated at room temperature in the dark for 30 min. Absorbance was measured at 517 nm. The ABTS radical cation was generated by reacting 7 mM ABTS with 2.6 mM potassium persulfate and incubating in the dark for 24 h. Then, 100 μL of the diluted sample was mixed with 900 μL of ABTS solution and incubated at room temperature for 30 min. Absorbance was measured at 734 nm.

The %*inhibition* for ABTS and DPPH radical scavenging activity was calculated as:

(2) $$\%inhibition=\frac{A_{Blank\;}-A_{Sample\;}}{A_{Blank\;}}$$

where *A_blank_* is an absorbance of blank solution after incubation; *A_sample_* is an absorbance of the sample.

ABTS and DPPH radical scavenging activities were determined using Trolox as the reference standard, with calibration curves constructed over 100–800 µM Trolox: DPPH, y = 0.001x + 0.012, R^2^ = 0.999; ABTS, y = 0.0013x − 0.0679, R^2^ = 0.999.

The bioaccessibility index (BI) was calculated for the digestion stages described above using a formula adapted from Andrade et al. [35] with the addition of a volume-based correction to account for stage-dependent dilution:

(3) $$BI\left(\%\right)=(C_{digesta}\times V_{digesta})/(C_{undigested}\times Q_{sample})\times 100$$

where C_digesta_ is the blank-corrected analyte concentration in the digesta (µmol TE. mL^−1^ for DPPH and ABTS; mg GAE L^−1^ for TPC); V_digesta_ is the total volume of the digestion fluid; C_undigested_ is the concentration measured in the undigested sample; and Q_sample_ is the quantity of sample introduced into the digestion vessel, expressed as mass (g) for the AGF sample and volume (mL) for liquid samples.

Dilution factors introduced at each digestion stage were incorporated into C_digesta_ and C_undigested_ prior to calculation of BI (%).

### 2.8. Determination of Physical Properties

Viscosity measurements were performed using a rotational viscometer DVPlus (AMETEK Brookfield, Middleborough, MA, USA) equipped with an LV-2 spindle at 37 °C with constant speed of 60 rpm. The viscometer was calibrated against a certified Newtonian viscosity standard before each measuring session. Viscosity values were expressed in mPa·s. Osmolality was measured using a OsmoTouch1 freezing point depression osmometer (Astori Tecnica s.r.l., Poncarale, Italy). The instrument was calibrated with standard solutions (100–1000 mOsm kg^−1^) prior to analysis. The pH of the samples was measured at room temperature using a pH/ion meter (Mettler-Toledo, Greifensee, Switzerland), calibrated with standard buffer solutions (pH 4.0, 7.0, and 10.0) before each measurement session. All three parameters were determined twice for each independently prepared replicate (*n* = 3) on the day following preparation.

### 2.9. Tube Flow Performance Evaluation

The flowability of the FBEA was evaluated using a gravimetrical enteral feeding system (Medetheren, Tel Aviv, Israel). A gravity feeding system was used with polyurethane tubes measuring 6 to 12 Fr (2.0 to 4.0 mm internal diameter) and 90 cm in length. Tubes were fixed vertically, and 200 mL of the product was passed through at room temperature (23.5 °C) from a height of 2 m. Commercial enteral formulas for comparison were selected to closely match the nutritional and physicochemical characteristics of the developed product.

The time required for complete passage of 200 mL was recorded using a stopwatch. A fresh gravimetric feeding system, consisting of a tube and reservoir bag, was used for each sample (*n* = 3 independent replicates). Tube occlusion and phase separation were monitored visually throughout the passage of the product, from the onset of flow through the tube until complete drainage. After 1 h, the tube and reservoir bag were inspected for sediment accumulation.

The flow rate was calculated using the following formula:

(4) $$Flow\;rate\;\left(\frac{mL}{min}\right)=\frac{Volume\;(mL)}{time\;for\;passage\;(min)}$$

### 2.10. Statistical Analysis

All experiments were performed on three independently prepared replicates (*n* = 3). Data were assessed for normality using the Shapiro–Wilk test and for homogeneity of variances using Levene’s test, with assumptions considered satisfied at *p* > 0.05 prior to parametric analysis. One-way ANOVA followed by Tukey’s Honestly Significant Difference (HSD) post hoc test was applied to compare group means at a significance level of α = 0.05. All statistical analyses and graphical visualizations were performed in R (version 4.5.2) using RStudio (Posit Software, PBC, Boston, MA, USA). Results are presented as mean ± standard deviation (SD).

## 3. Results and Discussion

### 3.1. Changes in Macronutrient Composition Under the Influence of Hydrolysis and Fortification

A comparative analysis of the macronutrient composition of AGF, AHS, CSA and developed FBEA, expressed on a dry weight basis (% DW), is presented in Table 2.

The main goal of enzymatic hydrolysis was to release macronutrient fractions into the liquid phase, while non-hydrolyzed components were transferred to the sediment and removed by centrifugation. This is consistent with current understanding of enzymatic modification of plant matrices, in which depolymerization of polysaccharide–protein complexes increases solubility and bioavailability [36]. Thus, hydrolysis should be interpreted not as a simple change in nutrient composition, but as a process of phase separation and selective solubilization of food matrix components.

The reduction in relative protein content from 14.84 ± 0.10% to 11.34 ± 0.11% and lipids from 6.54 ± 0.2% to 4.61 ± 0.2% in the AHS is primarily associated with co-precipitation of a portion of macromolecular complexes within the insoluble fraction, including denatured proteins, cell wall components, and lipid–carbohydrate associations. At the same time, the observed increase in carbohydrate content from 70.18 ± 0.96% to 77.42 ± 0.99% reflects hydrolysis of glycosidic bonds, where more monosaccharide units become available in the matrix compared to amaranth grain.

To isolate the contribution of the fortification blend from that of AHS, a formulation-matched control (CSA) was prepared by substituting AHS with distilled water, while all other ingredients remained unchanged. As CSA contains no amaranth-derived material, its macronutrient profile reflects the additive package alone. The protein content reached 31.82 ± 0.20%, markedly higher than in AGF and AHS, driven by the pea protein isolate. Lipid content similarly increased to 40.47 ± 0.93%, attributable to the safflower oil and sunflower lecithin emulsion. In contrast, carbohydrate content was correspondingly lower at 20.97 ± 1.95%, reflecting the smaller carbohydrate mass contributed by the additives relative to the native amaranth matrix.

A key technological step is the subsequent fortification of the AHS, during which the system acquires the properties of a restructured emulsion-dispersion matrix. The addition of protein isolate, lipid phase, and carbohydrate components leads to the formation of a structurally stabilized colloidal system in which macronutrients are distributed in the form of emulsified and associated complexes, which in turn guarantees the stability of the product.

The increase in protein up to 25.46 ± 0.50% and lipid content up to 24.95 ± 0.10% reflects not only the formulation-based addition of nutrients, but also their incorporation into a structured emulsion network. For protein, lipid, and carbohydrate content, FBEA values fell between AHS and CSA, indicating that the macronutrient composition of the final beverage results from the combined contribution of both the fortification blend and AHS. As described in Section 2.3, fortification was designed to meet target protein, lipid, and energy density levels for an isocaloric enteral formula, while AHS was retained as the functional base of the formulation. Fiber content did not differ significantly between AGF and FBEA, decreasing only in AHS, likely due to sedimentation during centrifugation. In contrast, CSA showed the highest fiber content among all samples, attributable to the stabilizer gum blend.

As a result, energy value increases from 491.74 ± 6.64 to 1014.7 ± 2.30 kcal L^−1^, which is explained both by redistribution of the lipid phase as the main energy carrier and by the increased total concentration of macronutrients in the FBEA. From a technological perspective, the developed process can be considered as a two-stage system: enzymatic hydrolysis followed by phase separation, resulting in a soluble and more bioavailable nutrient fraction and subsequent fortification leading to the formation of an emulsion system with defined nutritional value and stability. The observed isocaloric energy density and macronutrient distribution of the developed formula are generally consistent with the recommendations of the European Society for Clinical Nutrition and Metabolism (ESPEN) for enteral nutrition [21].

### 3.2. Amino Acid Composition, Distribution, and Adequacy After Hydrolysis and Fortification

#### 3.2.1. Characterization and Distribution of Free Amino Acids After Hydrolysis and Fortification

The free amino acid (FAA) profiles of AGF, AHS, and FBEA differed significantly across most of the 20 amino acids analyzed, reflecting the cumulative effects of enzymatic hydrolysis, centrifugal fractionation, and subsequent fortification with pea protein, as presented in Table 3.

Amaranth grain showed the lowest total free amino acid content among the three matrices, with particularly low concentrations of arginine at 0.52 ± 0.02 mg kg^−1^, lysine at 3.91 ± 0.68 mg kg^−1^, and histidine at 2.35 ± 0.14 mg kg^−1^. This result is consistent with the compositional characteristics of amaranth seed storage proteins, which are dominated by 11S globulins and glutelin, where these amino acids are present in peptide-bound rather than free form [37].

Tryptophan was the sole amino acid for which grain showed highest concentration at 328.69 ± 12.75 mg kg^−1^, since amaranth derives its protein primarily from water-soluble albumins, which house denser concentrations of tryptophan [38]. Conversely, it fell sharply from AGF to AHS, and then to FBEA, which likely reflects partitioning of free tryptophan into the insoluble pellet fraction during centrifugation.

Free glycine was not detected in AGF but was present in AHS and FBEA, most likely resulting from its release from the protein matrix during enzymatic hydrolysis [39].

After hydrolysis and subsequent fortification, an increase in the content of most free amino acids was observed. The most pronounced increases were noted for arginine, aspartic acid, glutamic acid, lysine, threonine, and methionine. These changes are likely attributable both to the breakdown of amaranth proteins with the release of bound amino acids, and to the contribution of pea protein, which is characterized by a high content of several of these amino acids [40].

Thus, the free amino acid profile of FBEA differed substantially from that of the untreated native grain, reflecting the combined effect of enzymatic hydrolysis, phase separation, and subsequent formulation fortification. These findings characterize the free amino acid composition of the developed formula but do not allow the individual contribution of the amaranth hydrolysate to these changes to be quantified, as CSA control was not included. Future work should extend amino acid profiling to CSA to isolate the contribution of AHS from that of the fortification blend.

#### 3.2.2. Essential Amino Acid Adequacy of FBEA

To assess the adequacy of the amino acid composition of the developed product, the content of essential amino acids was compared against the adult indispensable amino acid requirement patterns established by the WHO/FAO/UNU Expert Consultation (2007) [41]. The results of this comparison are presented in Table 4.

As shown in Table 4, the content of all seven essential amino acids in FBEA met or exceeded the reference values. Histidine content reached 117.9%, isoleucine 125.7%, leucine 135.1%, lysine 126.6%, and threonine 116.1% of the recommended levels. These values indicate that, for the amino acids assessed, FBEA has an adequate essential amino acid profile relative to the established physiological requirements of adults.

The smallest margin relative to the reference value was observed for valine, at 101.7%, whose content only marginally exceeded the recommended level. Considering the standard deviation of the measurements, the lower bound of the obtained range approaches the reference value. Therefore, despite formal compliance with the requirement, the adequacy of valine content in FBEA should be interpreted with some caution.

Amino acid composition in this study was determined using standard acid hydrolysis, the most widely applied method for the quantitative analysis of most proteinogenic amino acids. However, this approach has known limitations for tryptophan and the sulfur-containing amino acids, which require separate analytical protocols for accurate determination. Methionine was not detected in the analysis, while tryptophan and cysteine were outside the scope of the analytical method applied. Under standard acid hydrolysis, tryptophan is almost completely destroyed due to oxidative degradation. Methionine and cysteine also undergo oxidative degradation, forming methionine sulfoxide/sulfone and cysteic acid derivatives, respectively, as a result of reaction with residual oxygen during hydrolysis [42]. Accurate quantification of these amino acids requires additional steps. Methionine and cysteine require pre-oxidation with performic acid prior to acid hydrolysis. Tryptophan requires alkaline hydrolysis, for example, with sodium hydroxide [43,44].

The absence of data for these amino acids is therefore most likely due to methodological limitations of the sample preparation procedure used rather than their true absence from FBEA. This gap prevents calculation of a complete amino acid score and a definitive conclusion regarding the biological value of FBEA protein. Future studies should therefore apply the additional hydrolysis methods described above, or alternative analytical approaches less susceptible to acid-induced degradation. This would allow a complete essential amino acid profile to be obtained and support a more robust assessment of the amino acid completeness and protein quality of the developed formula.

### 3.3. In Vitro Bioaccessibility of the Phenols After Simulated Digestion

**Figure 1:**
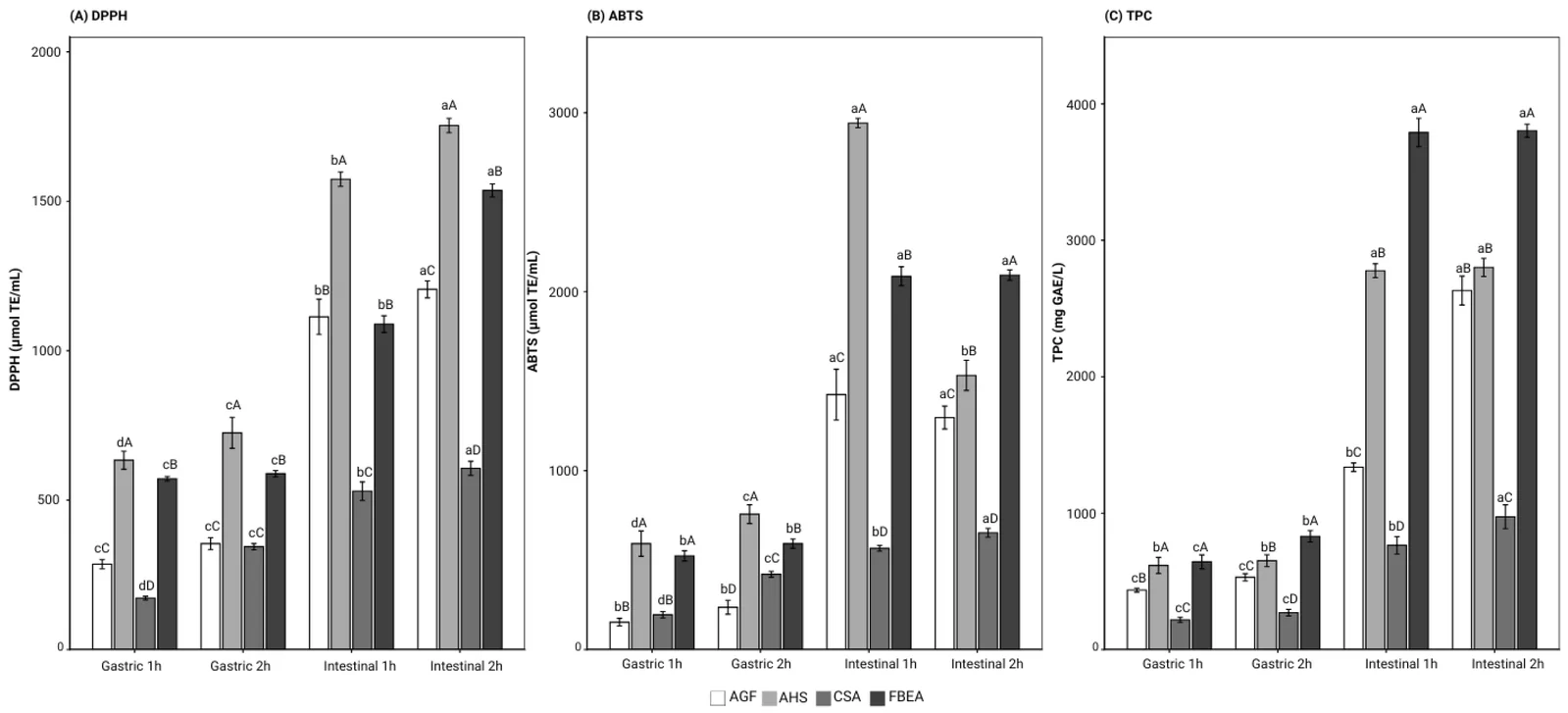
Bioaccessibility (%) of (**A**) DPPH radical scavenging activity, (**B**) ABTS radical scavenging activity, and (**C**) total phenolic content (TPC) of AGF, AHS, CSA and FBEA determined at four simulated in vitro gastrointestinal digestion time points (gastric phase, 1 h and 2 h; intestinal phase, 1 h and 2 h). Data are presented as mean ± SD (error bars), *n* = 3 independent replicates. The different lowercase letters above the bars denote significant differences (*p* < 0.05) among digestion stages for a given sample, whereas different uppercase letters indicate significant differences among the three samples at each stage. Bars sharing the same letter are not significantly different from one another. Values exceeding 100% indicate release of matrix-bound phenolic and antioxidant compounds that were not extractable from the undigested sample under the extraction conditions used rather than net synthesis or absolute absorption of these compounds.

In vitro bioaccessibility of phenols from AGF, AHS, CSA and FBEA was evaluated using the standardized INFOGEST digestion protocol (Figure 1).

The bioaccessibility of all measured parameters increased progressively from the gastric to the intestinal phase. During gastric digestion (1 h and 2 h), all four samples demonstrated a moderate increase in antioxidants and phenolics, characteristic of the acidic gastric environment, in which partial unfolding of proteins and release of bioactive components occur [45]. The intestinal phase, by contrast, substantially improved bioaccessibility across all samples. This pronounced increase is consistent with the synergy of pancreatic enzymes and bile salts at near-neutral pH, which promote matrix hydrolysis and the release of phenolic compounds previously retained within the food matrix structure [46].

AHS demonstrated significantly higher antioxidant bioaccessibility, as measured by DPPH and ABTS scavenging activity, than AGF, particularly following intestinal digestion, where AHS generally attained the highest antioxidant response among the four samples. This observation may be explained by prior enzymatic hydrolysis, which presumably increased the proportion of soluble, low-molecular-weight, and more readily digestible compounds, thereby facilitating their release during gastrointestinal digestion.

In contrast, CSA showed among the lowest DPPH and ABTS bioaccessibility of the four samples. At the gastric phase, CSA bioaccessibility was generally comparable to or lower than AGF, but at all stages, it remained significantly lower than both AHS and FBEA. This was observed despite CSA containing the same vitamin premix and other potentially reducing additives present in FBEA. This pattern suggests that the vitamin premix and remaining fortification components account for only a baseline level of antioxidant capacity, and that the substantially higher bioaccessibility observed in FBEA is attributed to amaranth-derived bioactive compounds released by enzymatic hydrolysis, rather than to the added commercial ingredients. These results suggest that the soluble fraction of amaranth grain has potential as an ingredient for improving the in vitro antioxidant profile of functional food products, although its physiological relevance beyond the in vitro assay would need to be confirmed in further studies.

FBEA was distinguished by high bioaccessibility of total phenolic content (TPC), particularly in the intestinal phase, where it showed the greatest phenolic response among the four samples. This indicates that digestion of the developed formula releases a greater proportion of bioaccessible phenolic compounds under the tested in vitro conditions. CSA similarly showed the lowest TPC bioaccessibility among the four samples at all digestion stages, supporting the idea that the fortification blend alone contributes minimally to phenolic release in the absence of the amaranth hydrolysate. AGF, by contrast, consistently ranked below AHS and FBEA across most parameters and at all stages of digestion, likely reflecting the intact structure of the native flour, in which the cell wall and compact protein–starch matrix restrict enzymatic accessibility and impede the release of bioactive compounds [47].

The bioaccessibility data obtained for FBEA may be of interest in the context of enteral nutrition. Enterally fed patients depend entirely on the formula to meet their nutritional requirements [48]. The extent to which compounds are released and solubilized during simulated gastrointestinal digestion can therefore inform expectations about their subsequent physiological availability in this population. Under the tested in vitro conditions, FBEA showed the highest intestinal bioaccessibility of phenolic compounds. For DPPH and ABTS scavenging activity, AHS generally showed the highest intestinal response, while FBEA remained the second-highest sample at most time points. This indicates that the beverage matrix does not restrict, and may even facilitate, the release of these compounds at the digestion stage most relevant to intestinal absorption.

Taken together, the increased bioaccessibility observed in FBEA appears to be driven predominantly by AHS rather than by the added fortification components. However, in vitro bioaccessibility reflects the release of compounds from the food matrix under simulated digestive conditions and does not, on its own, establish that these compounds are absorbed, reach systemic circulation, or confer an antioxidant or clinical benefit in vivo. It is therefore interpreted as favorable compound release under simulated digestion. Further validation using cellular uptake models and in vivo studies is warranted to establish the physiological and clinical relevance of these findings for enterally fed patients.

### 3.4. Dynamics of Physicochemical Parameters of the FBEA

**Figure 2:**
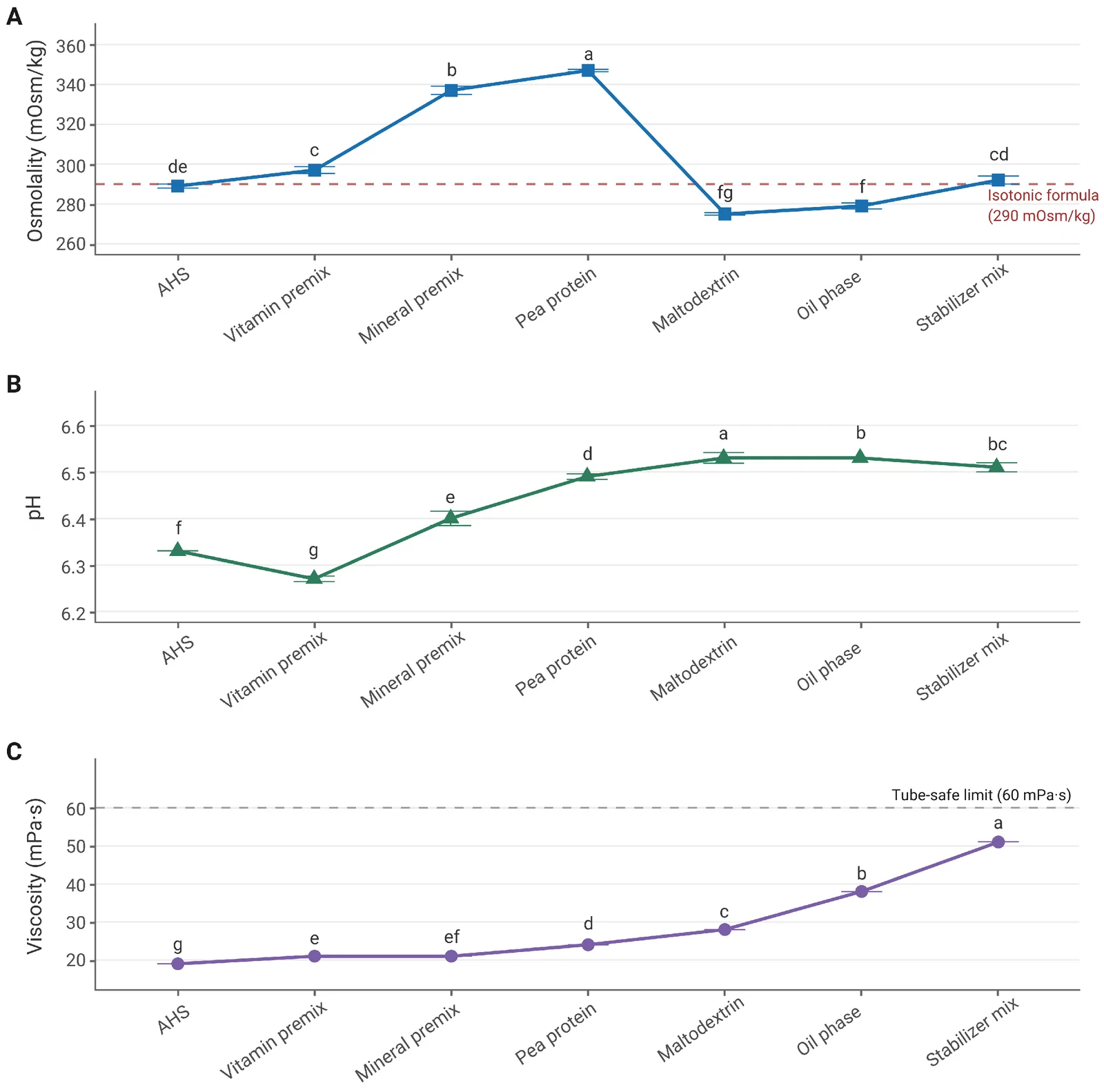
Stepwise changes in (**A**) osmolality (mOsm/kg), (**B**) pH, and (**C**) viscosity (mPa·s) of FBEA following sequential ingredient addition. The *x*-axis represents formulation steps in the order ingredients were added. Data points represent mean values, and error bars indicate the standard deviation of *n* = 3 independent replicates. Different letters above data points within the same panel indicate statistically significant differences between formulation steps (*p* < 0.05).

Despite nutritional regulations, the main requirements for enteral nutrition include a physiologically acceptable pH range (6.0–7.0), moderate osmolality (for isocaloric formulas 270–350 mOsm/kg), and viscosity that allows safe administration through a feeding tube without the risk of blockage [49,50]. The aim of the analysis was to monitor changes in osmolality, pH, and viscosity during the sequential addition of ingredients and to evaluate whether the developed formula meets physiological and technological requirements (Figure 2).

The initial osmolality of the AHS supernatant was 290 mOsm kg^−1^, reflecting the accumulation of osmotically active species, such as low-molecular-weight peptides, amino acids, and sugars generated during enzymatic hydrolysis [51]. Sequential addition of the vitamin and mineral premixes caused a moderate increase to 338 mOsm kg^−1^, primarily attributable to dissolved mineral ions. Subsequent dilution with maltodextrin solution reduced osmolality toward isotonic conditions, and incorporation of the lipid phase and stabilizer produced no further meaningful change. The final beverage osmolality of 292 mOsm kg^−1^ remained below the 400 mOsm kg^−1^ threshold considered safe for isocaloric enteral formulas [50].

pH values across all formulation stages remained within the near-neutral range of 6.27–6.53. A transient decrease to 6.27 following vitamin premix addition likely reflects the acidity of ascorbic acid and other organic acids present in the premix. The pH of FBEA remained within the acceptable range for enteral formulas [52].

Viscosity increased progressively with formulation complexity, from 19–21 mPa·s through the early stages to 51 mPa·s in the final product. Introduction of the protein fraction led to a moderate increase to 24 mPa·s, reflecting the hydrodynamic contribution of soluble proteins and peptides. The viscosity of the system grew to 28 mPa·s and 38 mPa·s after addition of maltodextrin solution and the lipid phase, respectively, indicating the formation of a structured dispersed system. Despite this gradual increase, the final viscosity remained below the 60 mPa·s thresholds considered safe for tube administration [53]. This is particularly important, as viscosity is recognized as a key factor influencing gastric emptying kinetics of enteral formulas [54].

Overall, the results demonstrate that the stepwise ingredient incorporation strategy enabled precise control of all three physicochemical parameters throughout the formulation process. The developed beverage met the ESPEN-recommended ranges for osmolality, viscosity, and pH, indicating suitability with respect to these specific physicochemical parameters for enteral administration. It is important to note, however, that these measurements reflect a single time point on freshly prepared samples. Longer-term behavior of the formulation—including phase separation, viscosity drift, pH change, lipid oxidation, vitamin degradation, and microbial stability during storage—was not assessed in the present study. The establishment of the shelf-life stability under these parameters therefore represents an important direction for future work.

### 3.5. Assessment of the Tubing Performance of the FBEA and Its Comparison with Commercial Formulas

To confirm the technological suitability of the FBEA for enteral delivery, its gravitational flow rate through a standardized feeding tube was determined and compared with those of commercial enteral nutrition formulas. The results are presented in Table 5.

The tubing performance of the fortified enteral formulation was comparable to that of the commercial enteral products, demonstrating appropriate flow characteristics and physical stability. The passage time of 200 mL of sample through the feeding tube was 465.67 ± 1.15 s, shorter than Commercial product 1 (505 ± 4 s) and slightly faster than Commercial product 2 (473.33 ± 4.16 s). Correspondingly, the flow rate of the fortified enteral product at 0.429 ± 0.001 mL s^−1^ was higher than that of both commercial comparators, but no statistical difference was observed with Commercial product 2, indicating favorable rheological properties for enteral administration.

The absence of sedimentation and tube occlusion in all three samples confirms the physical stability of the dispersed system and the homogeneous distribution of nutrients.

Overall, these results demonstrate that developed FBEA possesses rheological and physical properties fully compatible with routine enteral administration requirements and comparable to standardized commercial formulations.

The macronutrient profile of FBEA reflected its distinct formulation base. Protein content (5.07 ± 0.05 g/100 mL) fell between the two commercial products (4.02 ± 0.02 and 5.51 ± 0.12 g/100 mL), while total fat (5.04 ± 0.04 g/100 mL) exceeded both, and carbohydrate content (8.95 ± 0.12 g/100 mL) was lower than both. Fiber content was intermediate, and ash content was the lowest among the three formulations. The caloric value of FBEA was comparable to that of the commercial products. Overall, FBEA shows a comparatively higher lipid and lower carbohydrate contribution to total energy relative to the commercial comparators, consistent with its intended formulation strategy mentioned in Section 2.3.

FBEA exhibited the lowest osmolality among the three formulations—a favorable property for enteral formulations, as hyperosmolar formulas are associated with a higher incidence of gastrointestinal intolerance [48]. Viscosity was intermediate between the two commercial products and remained within a range compatible with standard feeding tube administration. pH was slightly lower than both commercial products but stayed within a physiologically acceptable range for enteral formulas [52].

## 4. Conclusions

This study demonstrated that the soluble fraction of enzymatically hydrolyzed *Amaranthus hypochondriacus* flour can potentially serve as a functional base for a plant-based enteral formula that meets key nutritional, physicochemical, and technological requirements for enteral administration.

The synergistic action of α-amylase and Alcalase hydrolyzed the amaranth grain matrix, significantly increased free amino acid content, with substantial increases in arginine (0.52 to 241.39 mg kg^−1^), lysine (3.91 to 129.10 mg kg^−1^), glutamic acid (103.62 to 368.54 mg kg^−1^), and GABA (33.45 to 244.04 mg kg^−1^). Fortification with pea protein isolate, safflower oil, and maltodextrin resulted in a dispersed emulsion system with 25.46% DW protein, 24.95% DW lipids, and 1014.7 kcal L^−1^, fulfilling the requirement for isocaloric formulas. When compared against the FAO/WHO/UNU reference amino acid requirement pattern, all seven essential amino acids in FBEA met or exceeded adequacy values, though the valine value should be interpreted with caution. Because protein digestibility was not assessed in this study, future work should target its assessment to determine the digestibility-corrected amino acid score of the developed prototype.

In vitro INFOGEST 2.0 digestion showed greater release of phenolic compounds and antioxidant activity from AHS and FBEA than from AGF, with FBEA achieving the highest intestinal phenolic recovery. Inclusion of a formulation-matched control (CSA) showed that CSA consistently exhibited the lowest bioaccessibility among the four samples, indicating that the enhanced antioxidant and phenolic release observed in FBEA is attributable primarily to the amaranth hydrolysate rather than to the added fortification components. As an in vitro measure, this indicates favorable compound release from the matrix but does not confirm absorption, systemic availability, or physiological benefit. Osmolality of 292 mOsm kg^−1^, pH of 6.51, and viscosity of 51 mPa·s fell within recommended ranges, and simulated tube-feeding showed flow comparable to two commercial formulas, with no occlusion or sedimentation.

Taken together, these findings support the technical feasibility of producing an amaranth-based enteral formula prototype with acceptable viscosity, osmolality, and tube flowability. However, clinical suitability cannot be concluded from these data alone; establishing it would require additional evidence on product safety, shelf-life stability, nutrient completeness, bioavailability, and patient tolerance, which are identified as directions for future work.

Moreover, the developed formula requires further evaluation against the regulatory and clinical requirements governing enteral nutrition products, including Technical Regulations TR CU 021/2011 “On Food Safety” and TR CU 027/2012 “On the Safety of Specialized Food Products,” GOST 35004-2023, and ESPEN/ASPEN guidelines. Subsequent work must confirm microbiological safety, sterility (ultra pasteurization or aseptic filling), endotoxin risk, and physicochemical stability over shelf life, together with levels of mycotoxins, heavy metals, pesticide residues, and plant-derived anti-nutritional factors, and compliance with allergen declaration, nutrient completeness, and labeling requirements. Clinical applicability ultimately requires preclinical and clinical studies confirming bioavailability, tolerance, efficacy, and safety.

## Figures and Tables

**Table 1:** Composition and functional role of additives in the FBEA.

Additive	Characteristics	Proportion Used for Fortification (g/100 mL)
Mineral premix	Premix for an isotonic-flavored beverage. Content at the recommended dosage per 100 mL: Sodium—51 mg; Potassium—13 mg; Magnesium—0.6 mg; Calcium—1.4 mg. Commercial food-grade mineral premix made in compliance with TU 10.89.19-005-51070597-2018.	0.165
Vitamin premix	Content: Antioxidant ascorbic acid (a source of vitamin C)—61.9 g; Niacin (nicotinamide)—15.9 g; Pantothenic acid (calcium D-pantothenate)—9 g; B6 (pyridoxine hydrochloride)—1.9 g; B1 (thiamine hydrochloride)—1.45 g; Folacin (folic acid)—0.27 g; B12 (cyanocobalamin)—1 mg; Food-grade vitamin premix produced in accordance with TU 10.89.19-005-51070597-2018.	0.019
Pea protein	Pea protein isolate. Nutritional and energy value per 100 g: Protein—82 g; Fats—4 g; Carbohydrates—3 g; Energy—380 kCal. Manufactured according to TU 10.89.19-014-24557090-2020 and compliant with TR TS 021/2011 [16], TR TS 022/2011 [17] and TR TS 029/2012 [18].	4
Maltodextrin	Produced in compliance with GOST 34274-2017, DE 20, E459.	3
Safflower oil	Unrefined oil of the first cold pressing. Does not contain food additives, flavorings, and is GMO-free, complying with ST RK 1428-2005. Produced under a food safety management system certified to ST RK ISO 22000-2019 [19] (ISO 22000:2018).	4.3
Lecithin	Sunflower lecithin, liquid, refined, E322. Produced under a food safety management system certified with FSSC 22000 [20] and ISO 22000.	0.77
Stabilizer mix	Food grade stabilizer–emulsifier blend containing mono- and diglycerides of fatty acids (E471), guar gum (E412), and xanthan gum (E415)).	0.77

Note: TU, GOST, ST RK, TR TS—Kazakhstan/Customs Union technical standards; ISO/FSSC—international food safety certification standards; DE, dextrose equivalent. Proportions are expressed as g additive/100 mL beverage.

**Table 2:** Nutritional profile (% DW) of amaranth grain flour (AGF), supernatant from amaranth hydrolysate (AHS), and functional beverage for enteral administration (FBEA), compared to control sample without AHS (CSA).

Component	AGF	AHS	CSA	FBEA
Protein	14.84 ± 0.10 ^b^	11.34 ± 0.11 ^a^	31.82 ± 0.20 ^d^	25.46 ± 0.50 ^c^
Total fat	6.54 ± 0.27 ^b^	4.61 ± 0.25 ^a^	40.47 ± 0.93 ^d^	24.95 ± 0.10 ^c^
Carbohydrates	70.18 ± 0.96 ^c^	77.42 ± 0.99 ^d^	20.97 ± 1.95 ^a^	44.17 ± 1.09 ^b^
Fiber	4.23 ± 0.11 ^b^	2.00 ± 0.12 ^a^	4.67 ± 0.15 ^c^	4.30 ± 0.09 ^b^
Ash content	3.99 ± 0.24 ^c^	2.98 ± 0.11 ^b^	1.87 ± 0.09 ^a^	2.16 ± 0.09 ^a^

Note: All values are expressed as percentage of dry weight (% DW). Different superscript letters within a row indicate statistically significant differences among samples (one-way ANOVA followed by Tukey’s HSD test, *p* < 0.05); means sharing the same superscript letter within a row are not significantly different. Results are expressed as mean ± standard deviation (SD), (*n* = 3 independent replicates).

**Table 3:** Free amino acid profile for AGF, AHS, and FBEA.

Amino Acid	Amount (mg kg^−1^ DW)		
	AGF (Control)	AHS	FBEA
Alanine	98.30 ± 2.76 ^a^	186.15 ± 5.62 ^b^	266.60 ± 5.64 ^c^
Arginine	0.52 ± 0.02 ^a^	241.39 ± 36.86 ^b^	511.80 ± 78.56 ^c^
Aspartic acid	130.38 ± 9.02 ^a^	288.69 ± 18.17 ^b^	555.91 ± 49.03 ^c^
Asparagine	14.03 ± 1.53 ^a^	102.26 ± 12.26 ^b^	139.96 ± 21.48 ^c^
GABA	33.45 ± 2.66 ^a^	244.04 ± 22.30 ^b^	317.99 ± 16.36 ^c^
Glutamic acid	103.62 ± 4.11 ^a^	368.54 ± 8.97 ^b^	516.38 ± 38.05 ^c^
Glutamine	134.09 ± 6.71 ^a^	167.84 ± 3.26 ^b^	294.63 ± 19.35 ^c^
Glycine	ND	62.00 ± 0.73 ^b^	72.72 ± 20.00 ^b^
Histidine	2.35 ± 0.14 ^a^	57.84 ± 0.13 ^b^	104.54 ± 0.14 ^c^
Isoleucine	122.18 ± 0.34 ^a^	119.57 ± 0.38 ^a^	192.56 ± 9.64 ^b^
Leucine	166.41 ± 0.97 ^a^	205.82 ± 3.78 ^b^	315.20 ± 1.98 ^c^
Lysine	3.91 ± 0.68 ^a^	129.10 ± 3.05 ^b^	174.32 ± 8.00 ^c^
Methionine	68.79 ± 0.61 ^a^	86.45 ± 1.19 ^b^	133.51 ± 2.25 ^c^
Phenylalanine	182.57 ± 3.13 ^b^	143.65 ± 1.96 ^a^	221.52 ± 5.24 ^c^
Proline	85.81 ± 0.36 ^a^	103.57 ± 1.11 ^b^	172.16 ± 2.74 ^c^
Serine	177.50 ± 3.62 ^a^	164.31 ± 8.43 ^a^	246.46 ± 6.14 ^b^
Threonine	75.55 ± 1.17 ^a^	103.28 ± 1.36 ^b^	172.93 ± 0.07 ^c^
Tryptophan	328.69 ± 12.75 ^c^	147.53 ± 2.66 ^a^	238.23 ± 3.43 ^b^
Tyrosine	66.54 ± 1.41 ^a^	146.59 ± 1.24 ^b^	236.08 ± 6.81 ^c^
Valine	190.89 ± 2.76 ^b^	161.81 ± 11.62 ^a^	253.13 ± 4.78 ^c^

Note: GABA, gamma-aminobutyric acid. Different superscript letters within the rows indicate significant differences according to Tukey’s HSD post hoc test following one-way ANOVA (*p* < 0.05). Results are expressed as mean ± SD (*n* = 3 independent replicates). ND—not detected and was excluded from the statistical comparison.

**Table 4:** Comparison of essential amino acid content in FBEA (mg/g protein) with reference adult amino acid requirement patterns.

Amino Acid	FBEA Content (mg g^−1^ Protein)	FAO/WHO/UNU Reference Pattern [41] mg g^−1^ Protein	Adequacy
Histidine	17.68 ± 0.79	15	117.9%
Isoleucine	37.71 ± 1.57	30	125.7%
Leucine	79.73 ± 12.57	59	135.1%
Lysine	56.95 ± 2.75	45	126.6%
Phenylalanine + tyrosine	54.59 ± 3.17	38	143.7%
Threonine	26.71 ± 3.14	23	116.1%
Valine	39.67 ± 3.14	39	101.7%

Note: Results are expressed as mean ± SD (*n* = 3 independent replicates) expressed as mg g^−1^ protein.

**Table 5:** Comparative tubing performance, macronutrient composition, and physicochemical properties of the developed FBEA and two commercial enteral nutrition formulas.

Parameter	FBEA	Commercial Product 1	Commercial Product 2
	Tubing performance		
Passage time of 200 mL, s	465.67 ± 1.15 ^a^	505 ± 4 ^b^	473.33 ± 4.16 ^a^
Flow rate, mL s^−1^	0.429 ± 0.001 ^a^	0.396 ± 0.003 ^b^	0.422 ± 0.003 ^a^
Presence of sediment	Not observed	Not observed	Not observed
Blockage of the tube	Not observed	Not observed	Not observed
	Macronutrient composition (g/100 mL)		
Protein	5.07 ± 0.05 ^b^	4.02 ± 0.02 ^a^	5.51 ± 0.12 ^c^
Total fat	5.04 ± 0.04 ^c^	3.60 ± 0.03 ^a^	3.72 ± 0.09 ^b^
Carbohydrates	8.95 ± 0.12 ^a^	12.94 ± 0.08 ^c^	11.32 ± 0.10 ^b^
Fiber	0.87 ± 0.02 ^b^	0.21 ± 0.01 ^a^	2.04 ± 0.02 ^c^
Ash content	0.64 ± 0.02 ^a^	1.20 ± 0.02 ^b^	1.35 ± 0.03 ^c^
Caloric value, kcal/100 mL	101.47 ± 0.23 ^b^	100.29 ± 0.13 ^a^	100.96 ± 0.45 ^c^
	Physicochemical properties		
Osmolality (mOsm kg^−1^)	292.0 ± 1.0 ^a^	301.0 ± 1.0 ^b^	331.67 ± 1.53 ^c^
Viscosity (mPa·s)	51.67 ± 0.15 ^b^	54.0 ± 1.0 ^b^	46.33 ± 0.57 ^b^
pH	6.51 ± 0.01 ^a^	6.93 ± 0.04 ^b^	7.32 ± 0.03 ^c^

Note: Commercial product 1 = “Nutrien Standard” (Infraprim LLC, Russia); Commercial product 2 = “Nutrison” (Nutricia, The Netherlands), as described in Section 2.1. Results are expressed as mean ± SD, *n* = 3. Different superscript letters within the same row indicate statistically significant differences among samples (Tukey’s HSD, *p* < 0.05).

## Data Availability

The original contributions presented in this study are included in the article. Further inquiries can be directed to the corresponding authors.

## Abbreviations

The following abbreviations are used in this manuscript:

INFOGEST	International Network on Food Digestion
EN	Enteral nutrition
ESPEN	European Society for Clinical Nutrition and Metabolism
FBEA	Fortified beverage for enteral administration
AHS	Supernatant from amaranth hydrolysate
AOAC	Association of Official Analytical Chemists
AGF	Amaranth grain flour
TPC	Total phenolic content
GAE	Gallic acid equivalents
DPPH	2,2-Diphenyl-1-picrylhydrazyl
ABTS	2,2′-Azino-bis (3-ethylbenzothiazoline-6-sulfonic acid)
BI	Bioaccessibility index
HSD	Tukey’s Honestly Significant Difference
ANOVA	Analysis of Variance
FAA	Free amino acid
GABA	Gamma-aminobutyric acid
